# Experience Since MELD Implementation: How Does the New System Deliver?

**DOI:** 10.1155/2012/264015

**Published:** 2012-10-02

**Authors:** Markus Quante, Christoph Benckert, Armin Thelen, Sven Jonas

**Affiliations:** Department of Visceral, Transplantation, Thoracic and Vascular Surgery, University Hospital Leipzig, Liebigstr. 20, 04103 Leipzig, Germany

## Abstract

Because of increasing waiting-list mortality, the MELD (Model for End-Stage Liver Disease) allocation system was implemented within most countries of the Eurotransplant area on December 16, 2006. Five years have now passed, and we review in this paper the effects of the MELD-based allocation upon the waiting list for liver transplantation, on peri-operative management and on postoperative outcome. Giving priority to sicker patients on the waiting list has resulted in a significant increase in mean MELD score at the time of organ allocation. Consequently, there has also been a significant reduction in waiting-list mortality. However, in Germany a worsening in postoperative outcome, mainly in the group of high-MELD recipients (≥30 points), has been reported. This paper presents comprehensive results following liver transplantation within the MELD era. Especially for the group of high-risk recipients, risk factors for impaired survival are presented and discussed.

## 1. Background

The last two decades have seen a steadily increasing number of patients on the waiting list for solid-organ transplantation. In the context of continual donor-organ shortage, this has engendered a persistent problem of allocation. As not every patient on the waiting list will be able to have timely organ access, the question of whom to serve first remains acute. Therefore, the existence of mandatory allocation rules is indispensable. When the German transplant law was enacted in 1997, the allocation system was based equally on cumulative waiting time and on individual clinical condition, as reflected by the Child-Turcotte-Pugh (CTP) score. This allocation principle was intended to serve the central demand for best utilization of each donor organ, in the sense of providing an optimum balance between medical urgency and postoperative outcome. However, throughout the years, waiting time became a major discriminating factor for patients on the waiting list; patients with acute liver decompensation, who were in urgent need of a transplantation but who had not accumulated enough waiting time, had a high risk of dying while on the waiting list, without receiving an organ offer. In addition, since 2002 there was an accelerated increase in numbers of patients on the waiting list for liver transplantation. These two factors led to a significant increase in waiting-list mortality [1].

Finally, on 16 December 2006 the MELD score was implemented as the basis for new allocation system in many countries within the Eurotransplant area. This model provides a prediction of 3-month mortality without liver transplantation. The score is calculated by combining bilirubin, creatinine, and INR (international normalised ratio) values only, and it therefore has an objective basis [2]. However, as there are many different possible underlying diseases that, despite chronic liver decompensation, often have only a modest impact on laboratory results, standard exceptions to the MELD system, with adjustment of the score, have been defined. For example, patients suffering from hepatocellular carcinoma are given an adjustment in their MELD score because of the underlying malignancy and the consequent anticipated tumour growth during the waiting period—risk factors that may not be reflected by laboratory results. Further common MELD exceptions are polycystic liver disease, cystic fibrosis, and metabolic disorders. In 2008—after first review and adaptation of the allocation system—also cholestatic liver diseases have been added to the standard exceptions due to the low impact of laboratory MELD in these patients. But the MELD exception for cholestatic liver diseases is only fulfilled in selected cases with septic complications. All diseases representing a standard exception according to the current guidelines defined by the German Medical Association and their initial adjusted MELD score is depicted in Table 1 [3]. The MELD-based allocation was intended to reduce waiting-list mortality by giving priority to cases with medical urgency. Five years after its implementation in the Eurotransplant area, there have been significant effects on waiting-list development, peri-operative management and postoperative outcome; these are presented and discussed in this paper.

## 2. Waiting-List Mortality

The implementation of the MELD allocation system has reflected a change towards an urgency-oriented model that was intended to improve organ access for sicker patients with higher MELD scores. At our centre, there was a significant reduction in waiting-list mortality from 18% in the year before to 10% in the year after MELD was introduced (*P* = 0.040) [4]. This result is in accordance with data already published on the 1-year mortality of patients on the waiting list for liver transplantation within the Eurotransplant area, comparing the period 2002–2005 (waiting-list mortality = 20%) with the year 2007 (only 10%) [5]. Other single-centre results within Europe have also confirmed a reduction in waiting-list mortality since the introduction of MELD [6]. Likewise, within the UNOS (United Network of Organ Sharing) area, where the MELD score was already implemented in February 2002, the introduction of MELD was followed by a steady decrease in waiting-list mortality and lower drop-out rates from waiting lists [7–9].

## 3. MELD Score at Time of Organ Allocation

After MELD implementation there was a significant increase in mean MELD score at the time of organ allocation, reflecting the intention to give priority to sicker patients on the waiting list. At our centre, the mean MELD score increased from 16.3 points in the year before to 22.4 points in the year after MELD introduction (*P* = 0.007) [4]. This observation accords with results from another single-centre analysis in Germany, where, again, a significant increase in mean MELD score at the time of liver transplantation, comparing the two years before and after MELD introduction, was observed (14.8 points versus 18.6 points; *P* = 0.001) [10]. Since then, there has been a steady increase in mean MELD score within the Eurotransplant area and especially in Germany. For September 2010, a mean MELD score of 34 points for standard organ allocation (without standard exceptions, without high-urgency status) was reported for Germany [5]. This trend is also reflected in the Eurotransplant Annual Report 2010, which describes a 24% increase in the number of high-MELD recipients (≥30 MELD points at the time of organ allocation) within the total population of liver-graft recipients in 2010 compared with 2009. In Germany in 2010, the proportion of high-MELD recipients among the total population of MELD-allocated liver transplantations was as high as 43% [11]. This might point to a specific problem in Germany, which has the highest proportion of high-MELD recipients compared with other Eurotransplant countries.

## 4. Donor Graft Quality

Since the adoption of MELD-based allocation there has been a significant increase in the numbers of donor grafts at our centre that fell within the scope of the extended donor criteria (EDC) as defined by the German Medical Association. In Germany, age distribution of organ donors showed a steadily increase of donors over 65 years during the last ten years. Finally, these donors accounted almost one-third of the organ donations in 2011 [12]. Hence, 30 percent of transplanted organs in 2011 already fulfilled EDC status only based on donor age irrespective of further medical details. As a consequence, the percentage of transplanted organs at our centre with deficiencies corresponding to at least one EDC increased from 24% in the year before to 60% in the year after the introduction of MELD (*P* = 0.001). In fact, there was an eightfold increase in the number of transplanted organs with deficiencies corresponding to two EDCs (*P* < 0.05) [4]. Retrospective validation of the donor risk index (DRI) [13] within the donor population in the Eurotransplant area between 2003 and 2007 showed that more than 50% of the transplanted organs had deficiencies corresponding to at least one EDC [14].

## 5. Postoperative Survival

Since the introduction of MELD-based allocation there have been several reports of impaired postoperative survival in Germany. The first publication describing reduced postoperative survival since MELD was a single-centre analysis, where the 90-day survival was only 79.6% in the two years after MELD compared with 88.6% in the two years before (*P* = 0.03) [10]. A German multicentre analysis including 462 patients confirmed the low 1-year survival rate of 75.8% one year after MELD. In particular, the group of high-MELD recipients (≥30 points) showed only the very poor 1-year survival rate of 53% [15]. At our centre, 90-day survival was found to remain stable at 90% when the year before and the two years after MELD were compared. There were also no significant differences in 1-year survival between different groups according to their MELD scores. The survival analysis showed a 1-year survival of 84% for the “MELD 6–19” group, compared with 81% for the “MELD 20–29” group and only 74% for the “MELD ≥ 30” group (*P* = 0.82) [4]. A comprehensive analysis of UNOS data for the ten-year period from 1997 to 2006 showed stable rates of patient and graft survival even after the introduction of MELD in 2002 [8]. In the clinical context of living donation within the UNOS area, survival analysis showed a favourable outcome even for high-MELD recipients (≥30 points), with a 1-year survival greater than 80% [16]. This might be due to the very strict patient selection that is practiced in connection with living donation. Therefore, we performed a retrospective analysis of our high-MELD recipients for factors predictive of impaired survival. This led us to the identification of the triad of dialysis, ventilatory support, and vasopressor administration within the 48 hours before liver transplantation as being highly predictive for reduced 1-year survival (30% for the triad group versus 86% for the control group; *P* < 0.001) [17].

## 6. Discussion

The implementation of MELD-based allocation has reflected a shift in allocation policy towards cases of medical urgency and has resulted in easier organ access for sicker patients, as reflected by a significant increase in mean MELD at time of organ allocation especially in Germany. In particular, a proportion of 43% high-MELD recipients with 30 or more MELD points represents the highest proportion compared to the most European countries. Although a comprehensive analysis of this development cannot be performed yet, there are several clinical factors which might contribute to this. On the one hand, the organ donor rate in Germany was only 15.8 per million population in 2010, which is ranked in the lower third compared to other European countries [12]. On the other hand, there has been a significant increase in donor organs fulfilling extended donor criteria in Europe during the last decade. In Germany, 30 percent of transplanted organs in 2011 already fulfilled EDC status only based on donor age irrespective of further medical details. As a consequence, the limited number of organs suitable for liver transplantation in the sickest waiting list candidates with 30 or more MELD points is de facto reduced since EDC grafts are not optimal to pair with high-risk recipients. Thus, persisting donor organ shortage in Germany is becoming more aggravated since there is a growing discrepancy between the increasing number of severe ill transplant candidates as intended by the “sickest-first” concept and the limited number of donor grafts that do not meet EDC.

Through this intentional prioritization of sicker patients, MELD has clearly been effective in reducing waiting-list mortality. On the other hand, there have been several reports of impaired postoperative survival since introduction of MELD. According to these analyses, the major contributory factor to impaired overall survival is a significantly worse survival in the group of high-MELD recipients (≥30 points) [10, 18, 19]. As a consequence, the possibility of imposing cut-off values for MELD score that might preclude transplantation because of increased postoperative mortality in these sicker patients has been discussed [18, 20]. 

However, comprehensive analyses have also been published that indicate no significant differences in survival between different MELD-score groups [4, 7, 21]. Furthermore, in the clinical context of living donation, favourable outcome in recipients even with 35 or more MELD points has been achieved [16, 22]. This emphasizes the importance of very strict patient selection, which is indispensable in the clinical setting of living donation. Nevertheless, as a consequence of the “sickest-first” concept implemented by the MELD allocation, the caveat must be made that it is probably not always possible to impose patient selection of a stringency comparable to that applied for living donation. Especially in high-MELD recipients, the decision on whether to accept an offered donor graft will often be difficult and confronts the physician with a dilemma of choice between hoping for a long-term advantage and avoiding an immediate risk. If a patient is in critical need of a liver transplant, and a donor graft of less than ideal quality is to hand, is it justified to postpone the transplant in the hope that a better organ might soon become available, while entailing the risk that it might not (with the consequent threat to the patient's short-term survival)?

Against the background of 43% high-MELD recipients among the total population of liver-graft recipients in Germany in the year 2010, close attention has to be paid to the analysis of risk factors for impaired survival in these severely ill patients [6]. Several approaches have been adopted to identify pre-operative parameters that predict postoperative outcome, in order to improve the allocation system in the sense of providing an equitable balance between medical urgency and ultimate outcome [23–25]. However, all these models seem to have their individual limitations [26]. At our centre, we follow strictly our simple clinical approach using the triad concept. With this policy we have been able to achieve a 1-year survival rate of 74% among our high-MELD recipients [17].

The triad concept is supported by recent published data from Munich on liver transplantation in patients with multiorgan failure (*n* = 18) [27]. Thirteen patients underwent transplantation directly from the Intensive Care Unit (ICU); ten of these 13 patients fulfilled the preoperative triad of dialysis, ventilatory support, and vasopressor administration. The 1-year survival was only 46% for these 13 patients from the ICU, which seems to be unacceptably poor. As a remarkable detail, the authors found that a decrease in MELD during the first 48 hours after admission to the ICU was associated with survival (*P* = 0.019). This finding also underlines the prognostic relevance of the first 48-hours interval after admission to the ICU. Clinical development during that period seems to determine whether substantial clinical amelioration can be achieved or whether the patient's clinical condition is in fact deteriorating, which in many cases would probably lead to exclusion from transplantation.

A further aspect that might have contributed to worsened survival since MELD implementation may have been the tendency to pair high-risk organs with lowest-risk patients (MELD 6–8 points). The increasingly ageing population—and consequently ageing donor organs—raises the question of optimal donor—recipient matching [28]. Analysis of US data from the SRTR (Scientific Registry of Transplant Recipients) showed a significantly higher mortality in lower-MELD recipients matched with high-risk donor grafts, whereas all recipients with MELD ≥ 20 points had a significant benefit from transplantation even when receiving high-risk donor grafts [29]. Other data from the UNOS registry also showed worsened posttransplant survival in less urgent patients (MELD <20 points), which was primarily attributed to changes in donor-organ quality [30]. These data provide clear evidence for the benefit of organ utilization by matching high-risk donors with sicker patients on the waiting list (MELD 20–29 points). At our centre, we adhere to this matching procedure. Thus, recipients with a MELD score of 20–29 received significantly more often organs meeting at least one EDC [4].

## 7. Conclusion

MELD has been effective in the reduction of waiting-list mortality by giving priority to sicker patients on the waiting list. However, in Germany, impaired postoperative survival rates after MELD implementation have also been reported. Detailed analysis suggests that this is mainly attributable to significantly worse outcomes in the group of high-MELD recipients, which in 2010 represented 43% of all liver graft recipients in Germany. Therefore, an extensive multicentric analysis of risk factors for impaired survival in these severely ill patients appears indispensable. Continuous review and adaptation of the allocation system will remain a challenge.

## Figures and Tables

**Table 1:** Standard MELD exceptions in Germany and their adjusted MELD score or adjusted 3-months-mortality, respectively [3].

Disease	Initial match MELD/adjusted 3-month mortality
Hepatocellular carcinoma (HCC)	15%
Hepatoblastoma	MELD 30
Polycystic liver disease	10%
Hyperoxaluria type 1	10%
Persisting dysfunction following OLT (including “small-for-size”)	Current lab MELD *plus * 20%
Cystic fibrosis	10%
Familial amyloid polyneuropathy (FAP)	15%
Hepatopulmonary syndrome	15%
Portopulmonary hypertension (POPH)	25%
Urea cycle disorders	MELD 30
Morbus Osler	15%
Hepatic epithelioid hemangioendothelioma (HEHE)	15%
Biliary sepsis/secondary sclerosing cholangitis (SSC)	Current lab MELD *plus * 30%
Primary sclerosing cholangitis (PSC)	15%
Cholangiocarcinoma	10%
